# MULTIPLE SMOOTH MUSCLE HAMARTOMA: CASE REPORT AND REVIEW OF THE LITERATURE

**DOI:** 10.4103/0019-5154.48993

**Published:** 2009

**Authors:** Ghaninezhadh Haydeh, Asgary Massoud, Noormohammadpour Pedram

**Affiliations:** *From the Department of Dermatology, Tehran University of Medical Sciences, Razi Hospital, Tehran, Iran*

**Keywords:** *Multiple*, *congenital*, *smooth muscle hamartoma*, *immunohistochemical*, *skeletal abnormalities*

## Abstract

Smooth muscle hamartoma (SMH) is a proliferative disorder of cells originating from muscle cells. It is a benign tumoral mass that usually presents as a single congenital skin-colored and hypertrichotic plaque involving the trunk and extremities. Multiple SMHs have rarely been reported in the literature. We describe the case of a seven-month-old girl with multiple SMHs located over the back and arm areas. The diagnosis was confirmed by biopsy and immunohistochemical (IHC) staining. She had no cerebral or skeletal abnormalities and her growth and development were normal.

## Introduction

A variety of cutaneous smooth muscle neoplasms may arise in the skin.[1] Smooth-muscle hamartoma (SMH) is an uncommon, usually congenital cutaneous hyperplasia of the arrectores pilorum muscles. When acquired, it may be confused with Becker's nevus with a prominent smooth-muscle component.[2] Smooth-muscle hamartoma was first described by Stokes in 1923.[3] Subsequently, in 1969, Sourreil, Beylot and Delfour reported congenital SMH (CMSH).[4] The lesions present as well-circumscribed, skin-colored plaques, frequently hypertrichotic, most commonly located on the trunk, buttocks or proximal extremities.[5] The estimated prevalence is about one per 2600 live births, with slight male predominance.[6]

Smooth-muscle hamartoma has been subdivided into two types, congenital (CSMH) and acquired (ASMH). The acquired type is distinct from the congenital form. CMSH is generally single, but multiple lesions have also been rarely reported in the literature.[6,7] A positive pseudo-Darier sign (temporary induration or piloerection after rubbing) is present in 80% of the patients.[6] Treatment of SMH is not necessary.[1,8]

## Case Report

**Figure 1:**
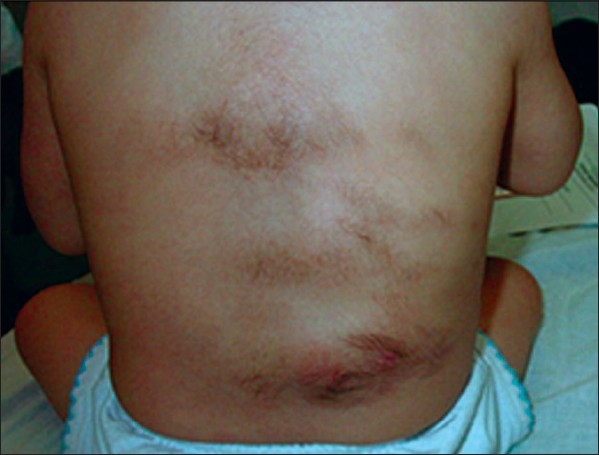
MSMH lesions on the back

**Figure 2:**
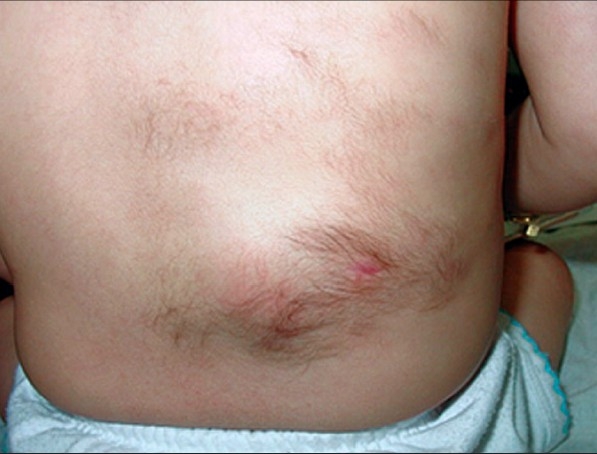
MSMH lesions, closer view

**Figure 3:**
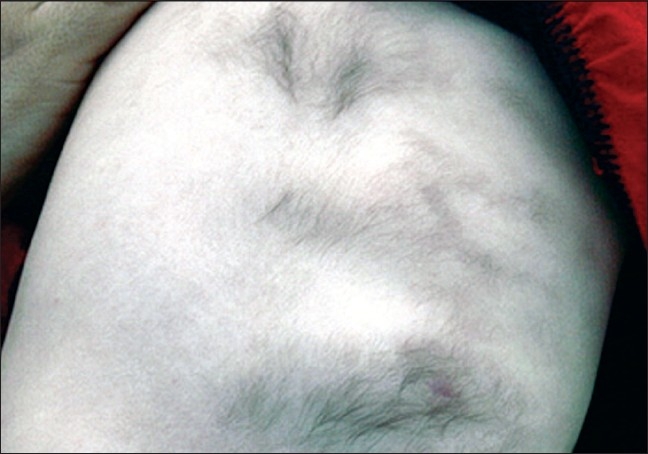
MSMH on the arm

A seven-month-old girl was evaluated for multiple lesions on the left mid-back and on the surface of the arms. Clinical examination found congenital multiple skin-colored, slightly raised plaques with hypertrichosis [Figures 1–3]. Hyperpigmentation was not present and the lesions were asymptomatic. A pseudo-Darier sign was observed after rubbing over some lesions.

**Figure 4:**
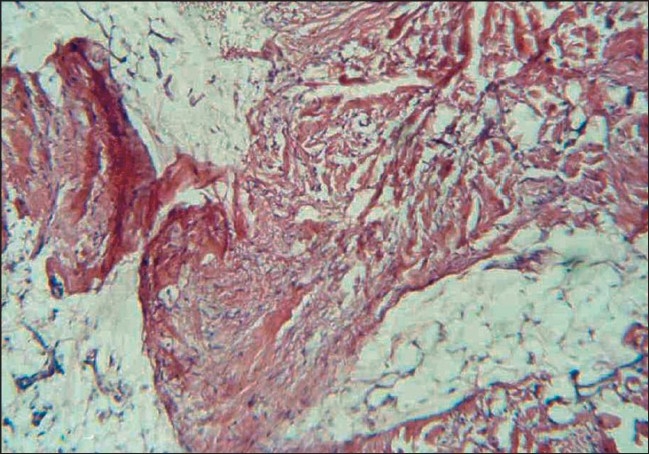
SMH pathology, H & E Staining (magnification ×400)

**Figure 5:**
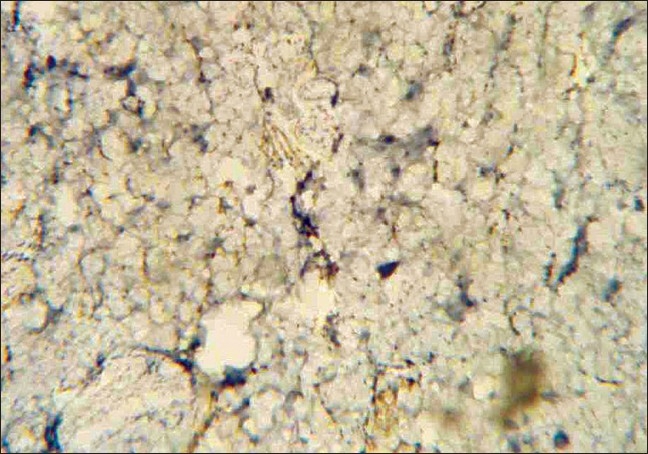
SMH IHC staining (magnification ×400)

**Figure 6:**
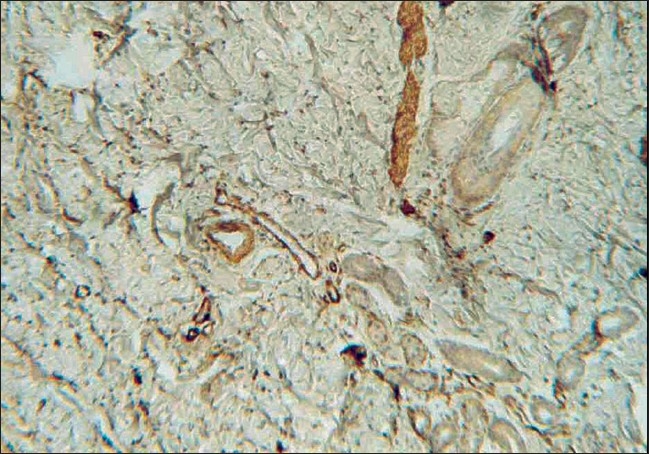
SMH IHC staining (magniÞ cation ×400)

Histopathologically, there were masses of spindle smooth muscle cells, distantly arranged in the superficial and deep dermis [Figure 4]. Immunohistochemical (IHC) stain with smooth muscle actin confirmed a diagnosis of SMH [Figure 5, Figure 6] and the nature of the proliferation. An MRI (magnetic resonance imaging) and CT (computerized tomography) scan showed no neurological deficit and abnormality in the central nervous system (CNS). Electromyography (EMG) and nerve conduction velocity (NCV) findings were also normal. The rest of the clinical examination was normal.

## Discussion

Hamartoma is derived from the Greek word *hamartanein*, which means “to err” or to “fail”. Hamartomas are defined as lesions that are (1) most commonly present at birth, but can also be acquired (2) composed of aberrant mature or nearly mature structures.[5]

Cutaneous smooth muscle hamartomas (SMHs) are caused by benign proliferation of smooth muscle bundle within the dermis. Congenital SMH (CSMH) is an uncommon malformation of the pilar smooth muscle that frequently involves the back and the lower limbs. Unusual cutaneous locations are the upper extremities, the face, and the mammary region.[9] Scrotal and eyelid-eyebrow involvement were also recently reported. Smooth muscle hamartomas present more frequently in male patients.[8,10]

In a review of 26 cases, 8% had prominent overlying hair, 12% were patches with peri-follicular papules, but no prominent hair, 61% were hyperpigmented, and 39% were skin colored.[11]

Rubbing of the plaque may result in a pseudo-Darier sign consisting of transient edema, erythema, and induration of the affected area, which may diminish in intensity with age.[12–14]

Most cases are congenital; a prevalence of up to 0.2% in children has been reported and has been increasingly recognized during the last two decades.[15] Most frequently, CSMHs are single; multiple lesions have been reported rarely.[7,9,27]

Familial cases have also been recently reported, but a great majority of the cases occur in a sporadic fashion.[16] Unusual clinical patterns have been reported in the literature, in particular multiple congenital lesions, or generalized forms with deep systemic involvement. A generalized variant with hypertrichosis and folding of the skin, called ‘Michelin Tire baby’ has been reported.[17,18] Congenital smooth muscle hamartoma (CSMH) with follicular spotted appearance is a rare clinical variant of CMSH in which the patients have marked peri-follicular papules in the patches. A linear distribution and a linear atrophic presentation of CSMH have also been reported.[19,20] Association with bilateral Becker's nevus too has been reported.[21] The clinical differential diagnosis of a single CSMH includes congenital melanocytic (pigmented) nevus, Becker's melanosis, solitary mastocytoma, pilo-leiomyoma, *cafe-au-lait* spots, and nevus pilosus and connective tissues nevi.[22] For multiple lesions, the main differential diagnosis is fetal alcohol syndrome.[7]

The histopathologic pattern is distinct from all other benign muscle tumors of the skin and is mimicked only by the smooth muscle hyperplasia present in Becker's nevus. Some authors consider these lesions as a part of a spectrum, while others prefer to keep them separate.[23]

The clinical lesions usually become less prominent with time and a review of the approximately 50 cases reported in the literature showed that there is no known associated systemic involvement or malignant transformation.[24]

There appears to be a rare acquired type of smooth muscle proliferation, which has been described under the title acquired SMH (ASMH).[8,25,26] The color of the lesions varies from pink to flesh tones to brown. They may be without hyperpigmentation or hypertrichosis. It has been reported most frequently in association with a Becker's nevus, but there are also reports of AMSH alone.[8]

Histologically, there is a marked increase of smooth muscle fibers in the dermis, especially in its deep portion. The fibers are grouped in sharply circumscribed bundles that are arranged haphazardly. They are not necessarily attached to hair follicles. There may be basal hyperpigmentation, as well as acanthosis and papillomatosis of the overlying epidermis.[16]

Histochemical stains such as Masson's trichrome and/or immunohistochemical (IHC) stains such as smooth muscle actin, muscle-specific actin, desmin and electron microscopic studies may confirm the smooth muscle nature of the proliferation.[1,18]

Clinical diagnosis of SMH is difficult, because of the lack of specific diagnostic criteria. The disorder must be suspected in any congenital hairy lesion, especially in the lumbosacral area.[1]

Treatment of SMH is not necessary, but involves surgical excision, if desired.[1,8,9,12]
